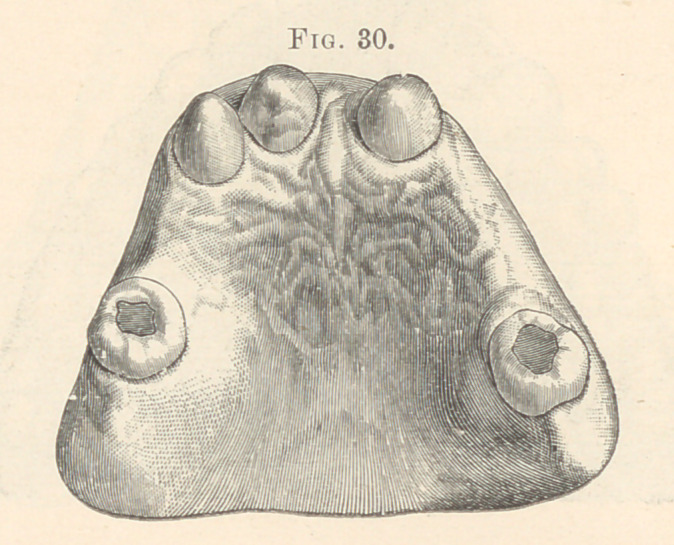# The Degenerate Jaws and Teeth

**Published:** 1897-02

**Authors:** Eugene S. Talbot


THE
### International Dental Journal.

Vol. XVIII. February, 1897. No. 2.

### Original Communications.[1]

### THE DEGENERATE JAWS AND TEETH.[2]

BY EUGENE S. TALBOT, M.D., D.D.S.[3]

Next to the ears, the jaws and teeth (as was to be expected from the variability of these organs in allied animals) are most affected by degeneracy. This is particularly true of the vertebrates, especially the mammals, as might have been anticipated from their phylogeny or line of descent. At the head of the vertebrates is man; at the foot is the lancelet (*amphioxus*), most akin to the semi-vertebrates the ascidians, who, in their larval phase, are higher than when adult, and whose life history excellently illustrates that potent phase of evolution, degeneracy.[4]

The lancelet[5] has a spinal cord inclosed in a soft semi-cartilaginous canal (the notochord). It is practically destitute of a brain. The cerebral vesicle which represents this is a plain cavity without true subdivision into ventricles. There is no cranium and the median eye is a mere pigment spot with which it is able to distin-

---

[1] The editor and publishers are not responsible for the views of authors of papers published in this department, nor for any claim to novelty, or otherwise, that may be made by them. No papers will be received for this department that have appeared in any other journal published in the country.

[2] Read in the Section on Neurology and Medical Jurisprudence at the Forty-seventh Annual Meeting of the American Medical Association, held at Atlanta, Ga., May 5 to 8, 1896. Reprinted from the Journal of the American Medical Association by special request.—[Ed.]

[3] Fellow of Chicago Academy of Medicine.

[4] Ray Lankester, Degeneracy, a Phase of Evolution.

[5] Willey, The Amphioxus.

guish light from darkness. Behind this is a small pit lined with cilia for olfactory purposes. Into this the cerebral vesicle of the larval lancelet opens. The mouth is well guarded against the intrusion of noxious substances, which have to pass through a vestibule richly provided with sensitive epithelial cells resembling the taste-buds of the human mouth. There is no heart. In this the lancelet is lower than the ascidians, the insects, crustacea, and many mollusks. It approximates the worms, which, despite a very elaborate vascular system, are destitute of a heart, the function of which is performed by contractile blood-vessels. From an embryologic and morphologic stand-point, the proximate ancestor of the vertebrates seems to have been a free swimming animal intermediate between an ascidian tadpole and the lancelet, while the primordial ancestor was a worm-like animal organized on a level with the starfish. The vertebrates, embryologically, develop from this stage to the lampreys, thence to the cartilaginous fish (shark), to the amphibia (frog, toad, axolotl), to the reptiles, and thence to the oviparous mammals (duckbill and spiny ant-eater), to the lemurs, and through forms like the pithecanthropus erectus to man. The present study will be confined to the mammals, passing from the simple types of teeth found in that oviparous edentate, the spiny ant-eater (echidna) of Australia to the indeciduous ancestors of the sloths and armadilloes and their descendants, inclusive of the dolphins and whales, whose teeth, both in fœtal Greenland and adult sperm-whale, preserve this old type. The whales,[1] it should be remembered, have degenerated from the hoofed mammals to suit their environment. While, as in the edentates, these teeth may be few, they may also (as in the insectivorous marsupials) approximate those of the reptilia in number (sixty or seventy on a side) and characteristic location.

The evolution of this primitive tooth to the bicuspid and molar type has been explained by two theories; the concrescence theory and the differentiation. The first, advanced by Magitot in 1877, was later advocated by Schwalbe, Carl Röse, and Kürkenthal. The last was offered by Osborn and Cope. Of these two contrasted theories Osborn[2] has given the following lucid presentation:

" Now let me illustrate, in a very simple manner, what is meant by the theory of concrescence and how we can imagine that the human molars have been built up by bringing together a number

---

[1] Haeckel, History of Creation, p. 242, vol. ii.

[2] INTERNATIONAL DENTAL JOURNAL, July, 1895.

of isolated teeth. Placing a number of conical teeth in line, as they lie in the jaw of the whale, they would represent the primitive dentition. In the course of time a number of these teeth would become clustered together in such a manner as to form the four cusps of a human molar, each one of the whale-tooth points taking the place of one of the cusps of the mammalian tooth,—in other words, by a concrescence, four teeth would be brought into one so as to constitute the four cusps of the molar crown. Vertically succeeding teeth might also be grouped. Now, what evidence is there in favor of this theory, and what is there against it? First, there is this, that all primitive types of reptiles from which the mammalians have descended and many existing mammals, as we have noted, have a large number of isolated teeth of a conical form; secondly, we find that by a shortening of the jaw, the dental fold or embryonic fold, from which each of the numerous tooth-caps is budded off in the course of development, may be supposed to have been brought together in such a manner that cusps which were originally stretched out in a line would be brought together so as to form groups of a variable number of cusps according to the more or less complex pattern of the crown. What may be advanced against this theory? This, and it is conclusive to my mind: We find at the present time that cusps, quite similar in all respects to each of the cusps which form the angles of the human molar, are even now being added to the teeth in certain types of animals, such as the elephant, whose molar teeth cusps are being complicated now or until very recent times. Then we find in the mesozoic period certain animals with tricuspid teeth. Now, according to the theory of concrescence these teeth ought not to show any increase of cusps in later geologic periods; but as we come through the ages nearer to the present time we find that the successors of those animals show a very much larger number of cusps. How is this increase of cusps to be accounted for? Has there been a reserve store of conical teeth to increase the cluster? No. Most obviously, to every student of the fossil history of cusps there is no reserve store, but new cusps are constantly rising up on the original crown itself by cusp addition. However, do not let me give you the impression that these researches of Röse and Kürkenthal are not of the greatest value and interest; we shall see later on how the very facts of embryology which are advanced by Dr. Carl Röse in support of his hypothesis can be turned against him and used to support the differentiation theory.

"Now let us turn to the differentiation theory and see what evidence we have of that. Let us go back to a very remote period of time, through the geologic ages of the pliocene and the miocene,

PLATE A.

through the eocene, through the cretaceous or chalk period, and even the jurassic. Still further back we go to the triassic, and the interval between this and the present period has been estimated at over ten million years. Now, in the triassic we find the mammalia, or the first animals which we can recognize as mammalia, possess conical, round, reptilian, or dolphin-like teeth. There are also some aberrant types which possess complex or multitubercular teeth.

"These teeth begin to show the first traces of cusp addition, as shown in the plate at the beginning of this article and in the accompanying key to this plate.

"Here (Fig. 1, Plate A) we have represented the teeth of the *dromatherium*, an animal found in this country in the coal-beds of North Carolina, and on the sides of the main cone are cusps or rudimentary capsules. In this enlarged model you see that on either side of the main cone are two cupsules. These teeth were

PLATE B.

found six hundred feet below the surface in a coal-mine, and in the same mine we find another animal, represented by a single tooth here (Fig. 2), in which these cusps are slightly larger. These cusps have obviously been added to the side of the tooth, and are now growing. Then we pass to teeth of the jurassic period, found in large numbers both in America and in England, but still of very minute size; and we observe the same three cusps, but these cusps have now taken two different positions; in one case they have the arrangement represented in Plate B; the middle cusp is relatively lower, and the lateral cusps are relatively higher; in fact, these cones are almost equal in size; these teeth are termed *triconodont*, as having three nearly equal cones. But associated with this of *triconodont* is another animal named *spalacotherium*, the teeth type of which are represented in Fig. 4. This is one of the most significant teeth which we have among all the fossil series, because this tooth illustrates the step that was taken in the transformation of a tooth (triconodont) with three cusps in line to a tooth with three cusps forming a *triangle;* for the primitive cusp is now seen to be the apex of a triangle, of which the two lateral cusps are the base. Now, this fact in itself is of great significance, because this tooth in this single genus is the key of comparison of the teeth of all mammalia of the great class to which man belongs. By this we are able, as you shall see, to determine that part of a human molar which corresponds with a conical reptilian tooth. The stage shown you is the triangle stage; the next stage is the development of a heel or spur upon this triangle, as you see in Fig. 5, *amphitherium.* To sum up: We have a reptilian cone, two cusps added to it, and a heel,—four cusps altogether,—and we shall now see what relation these bear to the human molar. First let us turn to some transitional forms. Examine a molar of the living opossum, a marsupial, which still distinctly preserves the ancient triangle. Look at it in profile, in side, or in top view, and see that the anterior part of that tooth is unmodified. This triangle we also trace through a number of intermediate types. In this figure (Fig. 6) of *miacis*, a primitive carnivore, we observe a high triangle and a heel, and when we come to look at it from above (Fig. 6*a*) we find that the heel has spread out broader, so that it is as broad as the triangle. Now, the three molars of this animal illustrate a most important principle,—namely, that the anterior triangular portion of the crown has been simply levelled down to the posterior portion of the crown. Compare these three teeth, therefore, and you see illustrated a series of intermediate steps between a most ancient molar and the modern molar of the human type. The second tooth is half-way between the first and third. Look at the second molar from above and you see it has exactly the same cusps as the first, so it is not difficult to recognize that each cusp has been directly derived from its fellow. Now direct attention to the third tooth of the series (Fig. 7), for it is of equal significance with the others. This tooth has lost one of its cusps; it has lost a cusp of the triangle. It is now a tooth with only half the triangle left on the anterior side, and with a very long heel. That tooth has exactly the same pattern as the lower human molar tooth (Fig. 8); the only difference is that the heel is somewhat more prolonged. These teeth belong to one of the oldest fossil monkeys, *anaptomorphus.* I have no doubt many of you have observed, in the examination of human lower molars that occasionally instead of having four cusps they have five. The fifth cusp always appears in the middle of the heel, does it not, or between the posterior lingual and the posterior buccal? You find this in the monkeys and in many other mammals, but I know of no record of the ancient anterior lingual reappearing. So we see that the human lower molar tooth with its low, quadritubercular crown has evolved by addition of cusps and by gradual modelling from a high-crowned, simple-pointed tooth."

Carl Röse[1] has contributed considerably to our knowledge of the evolution of the teeth. He says, "I find no mention in literature of the development of the teeth of the chamæleonidæ, nor of any other acrodont reptile. As the chameleon possesses multituberculate molars in the posterior portion of its jaws, therefore the development of the teeth in this animal must be doubly interesting, especially with regard to the origin of the molars generally. I examined the heads and jaws of both young and adult animals. Unfortunately, I was unable to procure embryos of the chameleon. All the material was sectionized into series of twenty-$\mu$ thickness, and doubly stained with alum carmine and bleu de Lyons. The figures have been drawn with Oberhäuser's camera. Fig. 13 shows

Fig. 13.

the teeth of the upper jaw five times magnified. The anterior teeth are unituberculate, the posterior ones bi- or trituberculate. All teeth are fused to the edge of the maxilla. *There is no shedding of the teeth in the chameleon*, nor could I prove it to take place in hatteria; but still there is, especially in the upper jaw, behind the functional teeth, a well-developed dental or reserve ridge. On its posterior end there takes place, throughout life, a continuous new formation of teeth. Accordingly, older animals have always a larger number of teeth than young ones. Although I examined macroscopically, with a lens, a number of heads of the chameleon, and microscopically six different series of sectionized jaws, I never succeeded in finding any indications of reserve teeth."

---

[1] Dental Cosmos, October, 1893.

To alienists, biologists, criminal anthropologists, and sociologists the human jaw and teeth are of peculiar interest, since their study establishes many points in evolution and environment not clearly determinable in other structures. Their study enables the observer without much difficulty to determine inherited and acquired stigmata. For this purpose the teeth should be studied from the first evidence of their development until they are all in place, which occurs normally in most cases by the twenty-second year.

Enamel of the teeth is formed from the epiblast, and dentine, cementum, and pulp (except as to nerve-tissue) from the mesoblast. The enamel organs of the first set form during the seventh week of fœtal life, the dentine bulb during the ninth week. At this period the tooth obtains its shape and size, and calcification begins at its periphery. This models the enamel cap, which fits over the dentine like a glove. When imperfections in hand or fingers exist these deformities are distinctly observed upon the glove. In precisely the same manner are observed the different shapes and sizes of the incisors, cuspids, and molars. Calcification of the teeth begins at the seventeenth week of fœtal life. Illustration (Fig. 14)

Fig. 14.

shows the progress of calcification and development of the temporary set of teeth. Examination will show that any defect in nutrition from conception to birth (due to inherited states or maternal impressions) has been registered upon the teeth. The state of the constitution and the locality registers the date of such defects. Thus, if the tooth as a whole be larger or smaller than normal, or abnormally irregular, taint is undoubtedly inherited from one or both parents. If, on the other hand, there be defect at any part on the crowns of the teeth, and the contour be perfect, the date of malnutrition can be easily determined by consulting this chart. More or less than the normal number of teeth abnormally placed

FIG. 15.

12 years
10 ,,
9 ,,
8 ,,
7 ,,
6 ,,
5 ,,
4 ,,
3 ,,
2 ,,
1 ,,
At birth
20
18
16
14
12
10
9 8
25th week.
embryo.

demonstrate the existence of inherited defect, since the germs must have been deposited at the periods mentioned. No absolute

Fig. 16.

Fig. 17.

Fig. 18.

Fig. 19.

rule can be laid down as to the date of the eruption of the teeth. The teeth of the temporary set erupt nearly as follows:

| | After Birth. | Time of Eruption. |
|---|---|---|
| Lower central incisors . . . . . | 7 months. | 1 to 10 weeks. |
| Upper central incisors . . . . . | 9 months. | 4 to 6 weeks. |
| Upper and lower lateral . . . . | 12 months. | 4 to 6 weeks. |
| First molars . . . . . . . . . | 14 months. | 1 to 2 months. |
| Cuspids . . . . . . . . . . . | 18 months. | 2 to 3 months. |
| Second molars . . . . . . . . | 26 months. | 3 to 5 months. |

The enamel organs and dentine bulb for the permanent teeth form just before birth (Fig. 15) in like manner with the temporary set. They form just above the temporary set on the upper and below on the lower jaw. The permanent molars begin to calcify at the twenty-fifth week of fœtal life. The permanent incisors do not calcify until a year after birth. Any deviation in size or contour of the permanent teeth from the normal must hence be due to defect in nutrition in the dentine bulb between the fifteenth and twenty-fifth week of fœtal life. Any deviation in calcification (ex-

Fig. 20.

cept the cusps of the first permanent molars) must occur after birth. At the third year twenty-four teeth are fairly well calcified. At the fifth year the second permanent molars and at the eighth year the third molars or wisdom teeth begin to calcify.

The following table gives the age of eruption of permanent teeth:

| | |
|---|---|
| First permanent molars . . . . . . . . . . . . | Circa 6 years. |
| Upper and lower central incisors . . . . . . . . | Circa 7 years. |
| Upper and lower lateral . . . . . . . . . . . | Circa 8 years. |
| First bicuspids . . . . . . . . . . . . . . . | Circa 9 years. |
| Second bicuspids . . . . . . . . . . . . . . | Circa 10 years. |
| Cuspids . . . . . . . . . . . . . . . . . . | Circa 11 years. |
| Second permanent molars . . . . . . . . . . . | Circa 12 years. |
| Third permanent molars . . . . . . . . . . . | Circa 17 to 24 years. |

Man at his present stage of evolution has twenty teeth in his temporary and thirty-two in his permanent set. Any deviation in number is the result of embryonic change occurring between the sixth and fifteenth week for the temporary teeth and the fifteenth week and birth for the permanent. The germs of teeth which erupt late in life and are (properly) called third sets, of necessity appear ere birth, and are completely formed at the beginning of the second year, although they remain protected in the jaw until late in life.

More than twenty teeth in the temporary or than thirty-two in the permanent is hence an atavistic abnormality.

From a maxillary and dental stand-point man reached his highest development when his well-developed jaws held twenty temporary and thirty-two permanent teeth. Decrease in the numbers meant, from the dental stand-point, degeneracy, albeit it might mark advance in the man's evolution as a complete being. Marsh[1] points out that in the New Mexican lower eocene occur a few representatives of the lowest primates, such as the lemurarius and limnotherium, each the type of a distinct family. The lemurarius, most nearly allied to the lemurs, is the most generalized primate yet found. It had forty-four teeth in continuous series above and below. The limnotherium, while related to the lemurs, had some affinities with the American marmosets. Dr. A. H. Thompson,[2] in discussing the "missing teeth" of man, remarks that these researches of Marsh suggested and subsequent studies aided the solution of the problem of the origin of the extra teeth (known as supernumeraries) that sometimes occur in man. These, usually regarded as pure freaks, like polydactylism, are, however, beautiful illustrations of atavism, and demonstrate that man during his evolution from the lowest primate has lost twelve teeth. These supernumerary teeth assume two forms,—either they resemble the adjoining teeth or are cone-shaped. While they rarely are exactly counterparts, every tooth can be and is duplicated, as the following illustrations show. Fig. 16 illustrates fairly well-formed duplicate central incisors, the normal incisors being outside the dental arch. They are crowded laterally by the large roots of the supernumerary incisors. Fig. 17 shows an extra right lateral in a temporary set in the upper jaw; Fig. 18, an extra right lateral in the permanent set. Fig. 19 illustrates normally developed supernumerary cuspids, which are all

---

[1] Vertebrate Life, in American Association for Advancement of Science, 1877.

[2] Dental Cosmos, 1894.

grouped together upon the right side, the bicuspids being also duplicated on each side; indeed, all but the molars are duplicated. Fig. 20 shows supernumerary third molars easily demarcated from the normal molars. The teeth, which fail to approximate their normal neighbors, assume the cone shape of the primitive tooth.

The fact that the cone-shaped tooth, as a rule, is perfect in construction, is found everywhere in the jaw, but especially in the anterior and posterior part of the mouth, is of much value in outlining tooth and jaw evolution, especially from degeneracy aspects. The upper jaw, being an integral part of the skull and fixed, is of necessity influenced by brain and skull growth, hence degeneracy is more detectable in it than in the lower.

The evolution of the jaw is towards shortening in both directions. This shortening will continue so long as the jaw must be adjusted to a varying environment. The jaw of man having originally contained more teeth than at present, lack of adjustment to environment produces from the shortening degeneracy of the jaw and atavism of the teeth. While this may coincide with general advances of the individual, it indicates that he is not yet adjusted to his new environment. The shortening of the upper jaw causes supernumerary cone-shaped teeth to erupt in mass at the extreme ends of the jaw, as shown in the following figures. Fig. 21 illustrates a cone-shaped tooth between the two central incisors, forcing them out of position. Fig. 22 shows three supernumerary teeth; a cone-shaped tooth between the central, lateral, and cuspids out of position. The left permanent lateral is at the median line, another cone-shaped tooth remains in the vault, while the supernumerary left lateral is in place. As many as eight are at times to be observed in the anterior vault. Posteriorly these teeth are most often noticed in connection with third molars, usually on a line with other teeth, posterior to the last molar. Fig. 23 shows two supernumerary cuspids in the anterior and two in the posterior part of the left arch; the molars have been extracted. Supernumerary teeth are not confined to these localities, but may be observed at any point in the dental arch. (Figs. 24 and 25.) The primitive cone-shaped tooth is rarely observed in the lower jaw. In twenty-six years' practice I have not seen a case. The mobility of the lower jaw prevents that maladjustment to environment present in the upper.

The continual shortening in both directions of the jaw causes the third molars frequently so to wedge in between the angle of the jaw and the second molar that eruption, if possible, is difficult.

Fig. 21.

Fig. 22.

Fig. 23.

Fig. 24.

Fig. 25.

Fig. 26.

Fig. 27.

Fig. 28.

Fig. 29.

Fig. 30.

The third molar is often absent in the Caucasian races. In forty-six per cent. of six hundred and seventy patients it was missing. Frequently its development is abortive. This tooth in the struggle for existence seems destined to disappear. It is more often absent from the upper than the lower jaw. When absent or badly developed the jaw is smaller, and frequently teeth irregularities, nasal stenosis, nasal bone and mucous membrane hypertrophy, adenoids, and eye disorders coexist. Fig. 26 shows absence of the left third molar, with irregularities of that side of the arch. In Fig. 27 both third molars are seen to be missing, coincident with irregularities on both sides of the arch. Anteriorly the lateral incisors are most often wanting; fourteen per cent. of the laterals were wanting in six hundred and seventy patients. In the progress of evolution man has lost one lateral upon each side of the mouth, and the second lateral seems also destined to disappear. In Fig. 28 the left lateral incisor has disappeared, and in Fig. 29 both lateral incisors are absent. Not infrequently does it occur that centrals, cuspids, bicuspids, and even molars are absent; even their germs are not detectable. Fig. 30 illustrates a cast showing three supernumeraries in the anterior part of the mouth and but two molars. The absence of teeth indicates lack of development of germs, due either to heredity or defective maternal nutrition of the line of conception or during early pregnancy.

(To be continued.)

## Figures and Tables

**PLATE A. f1:**
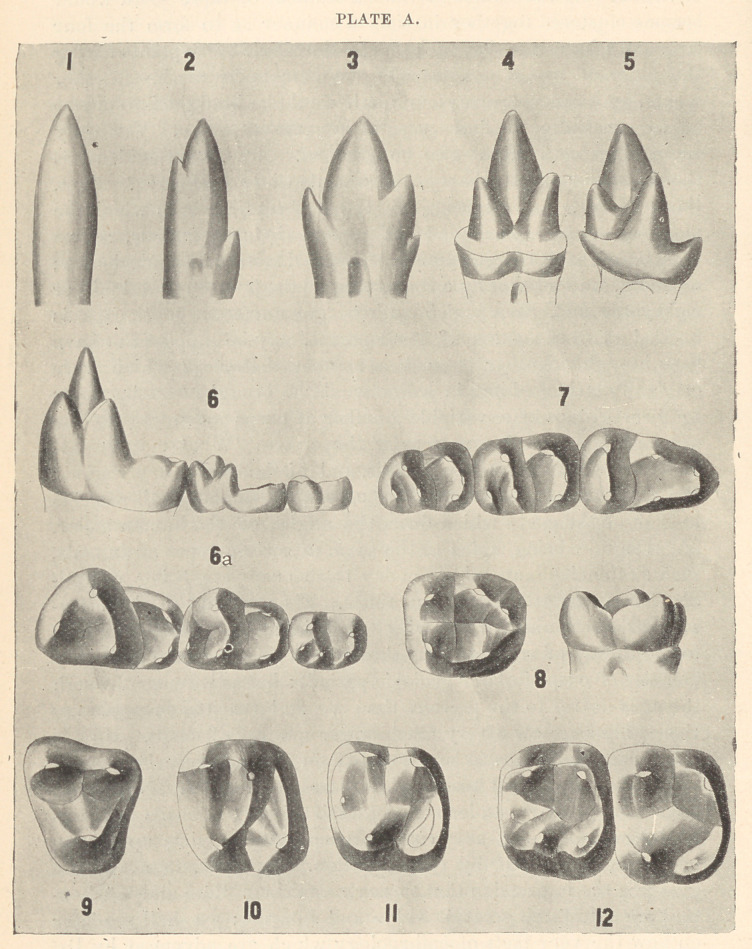


**PLATE B. f2:**
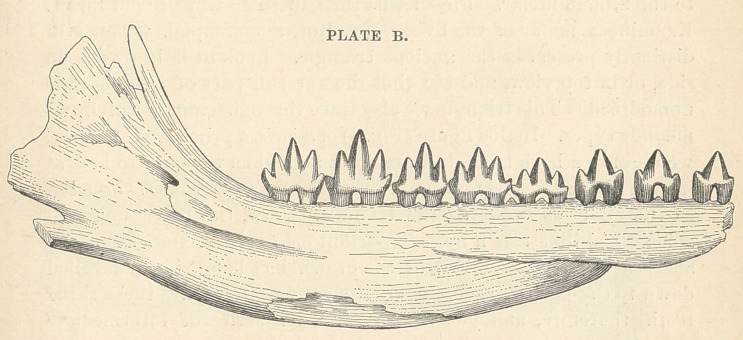


**Fig. 13. f3:**
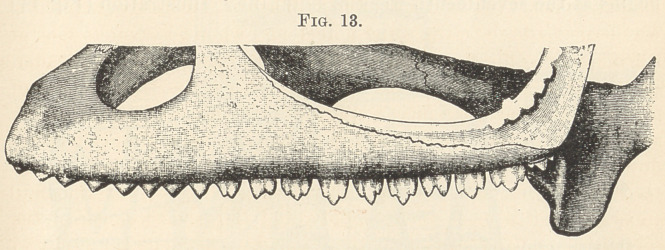


**Fig. 14. f4:**
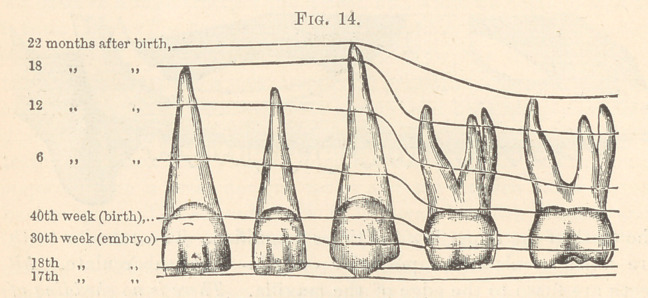


**Fig. 15. f5:**
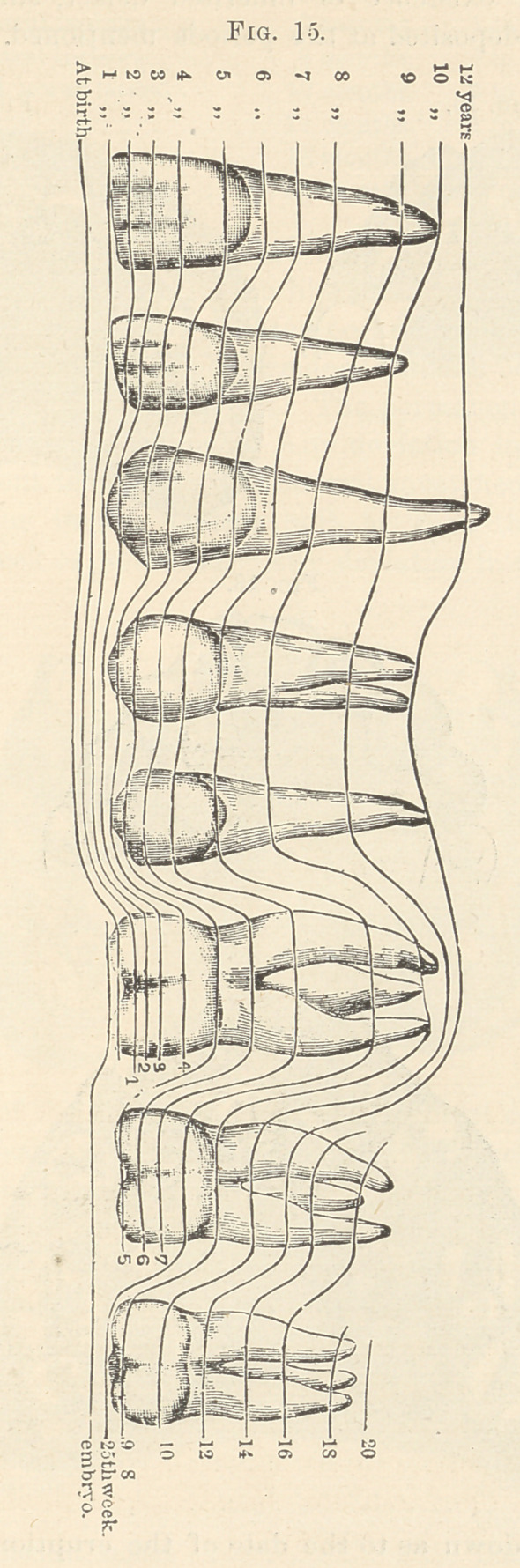


**Fig. 16. f6:**
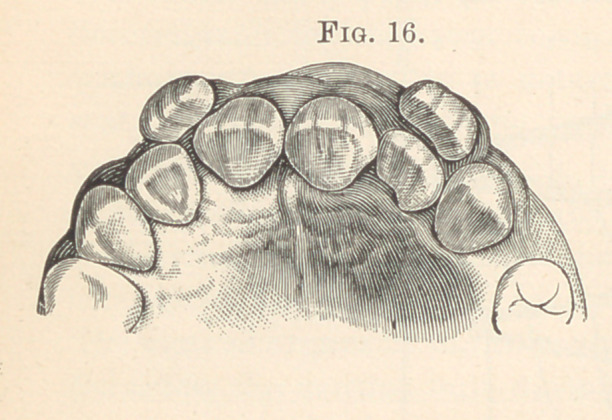


**Fig. 17. f7:**
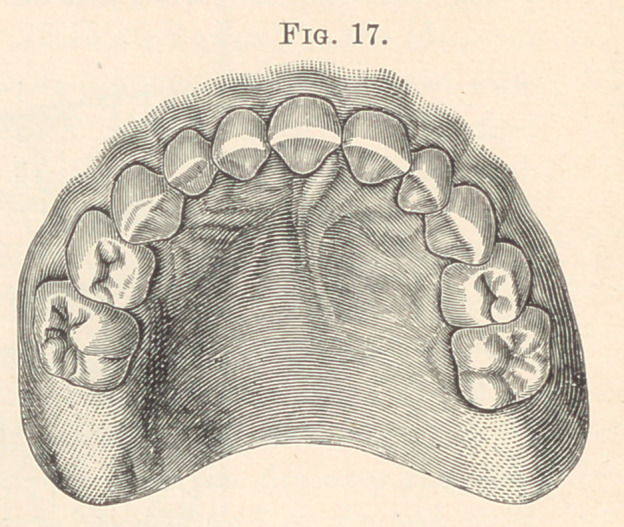


**Fig. 18. f8:**
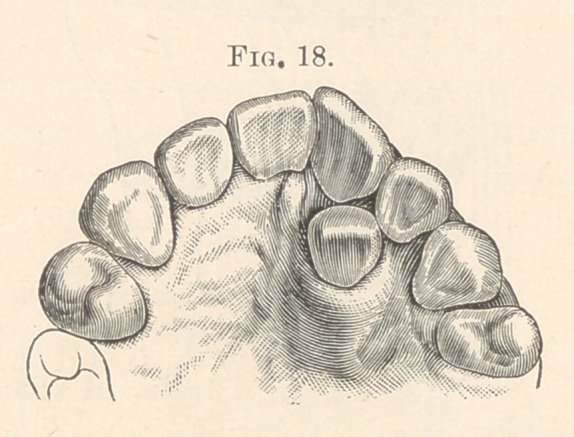


**Fig. 19. f9:**
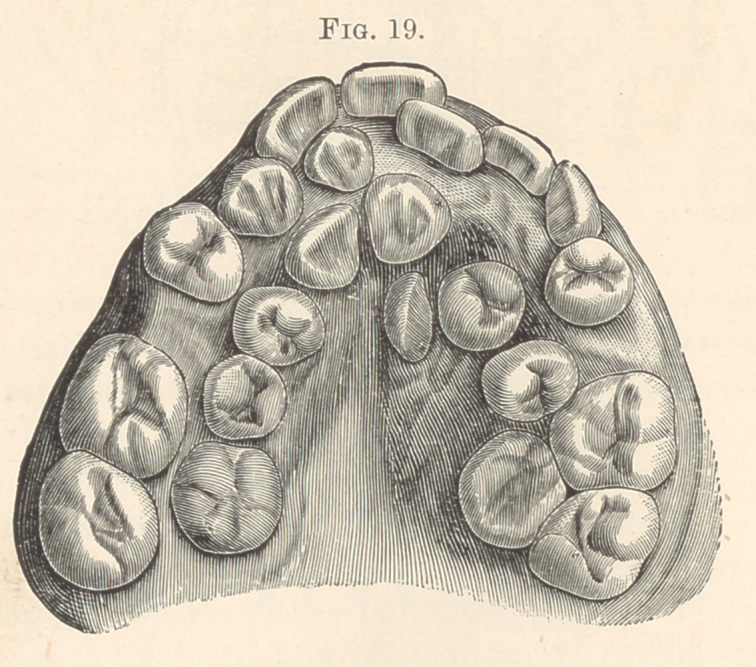


**Fig. 20. f10:**
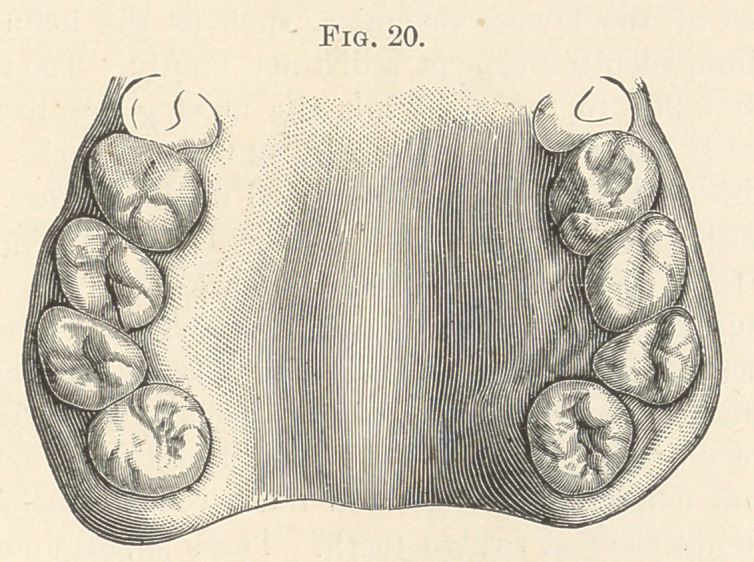


**Fig. 21. f11:**
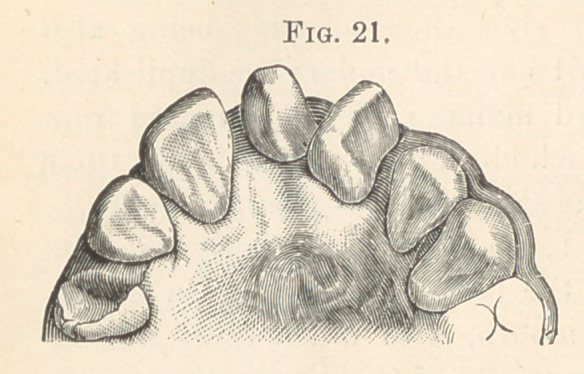


**Fig. 22. f12:**
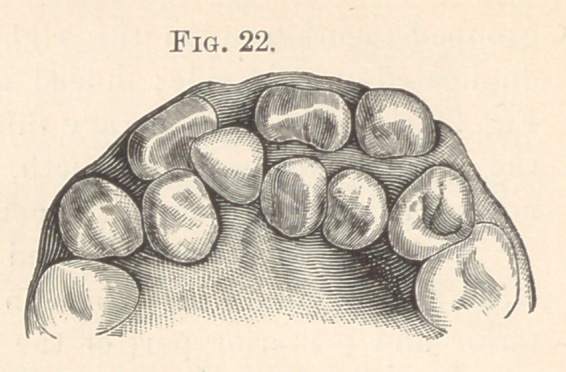


**Fig. 23. f13:**
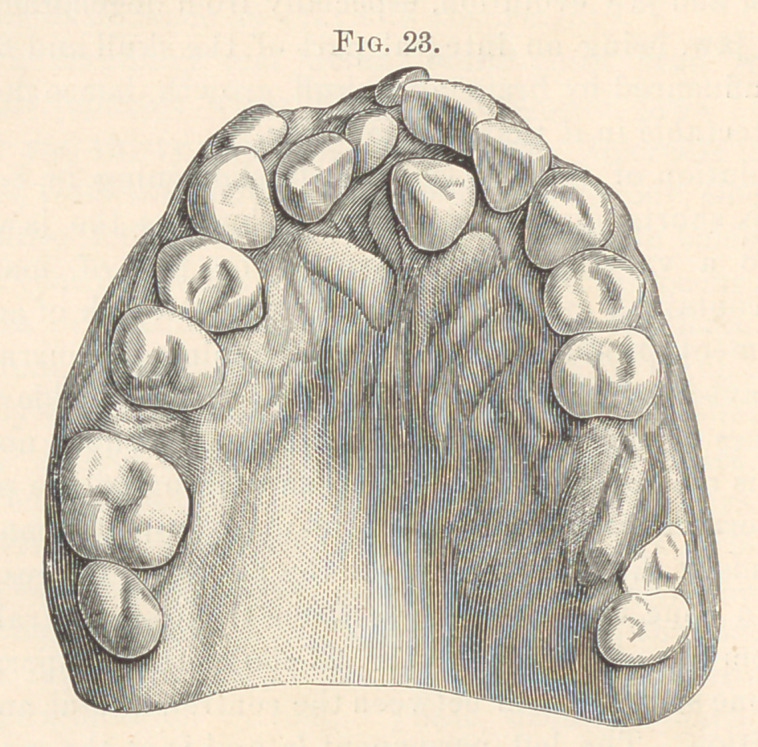


**Fig. 24. f14:**
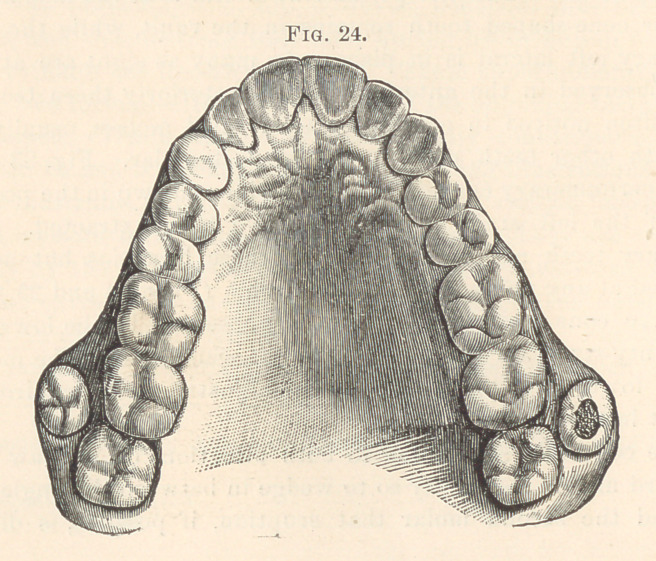


**Fig. 25. f15:**
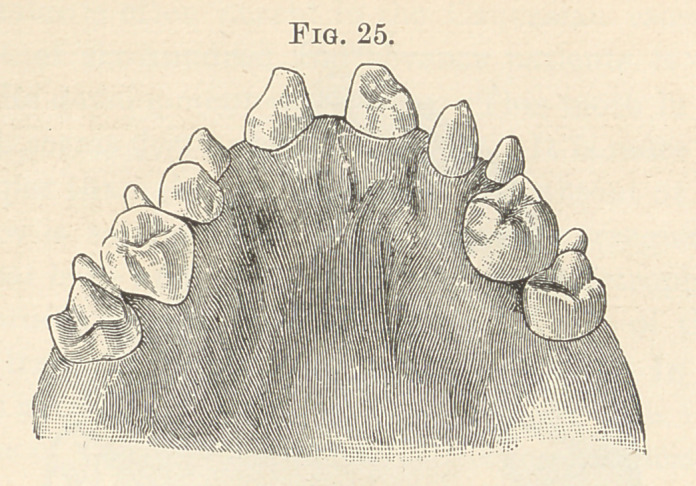


**Fig. 26. f16:**
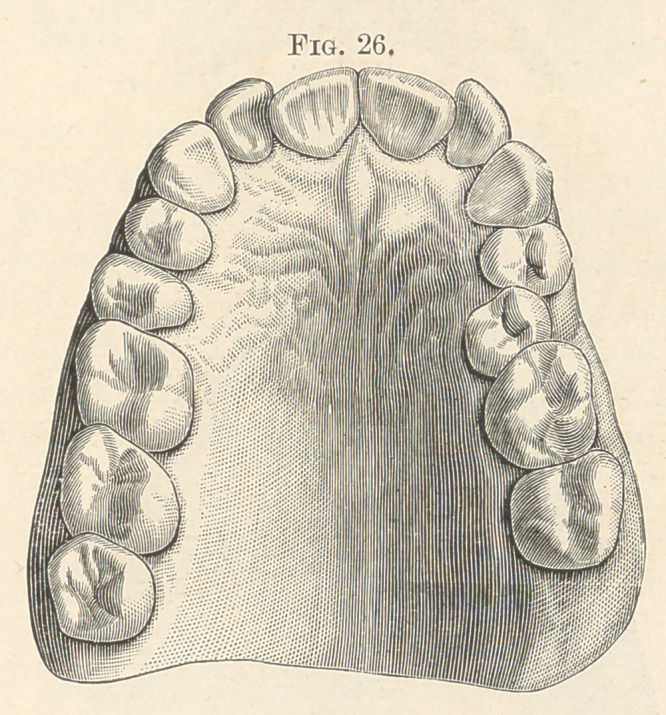


**Fig. 27. f17:**
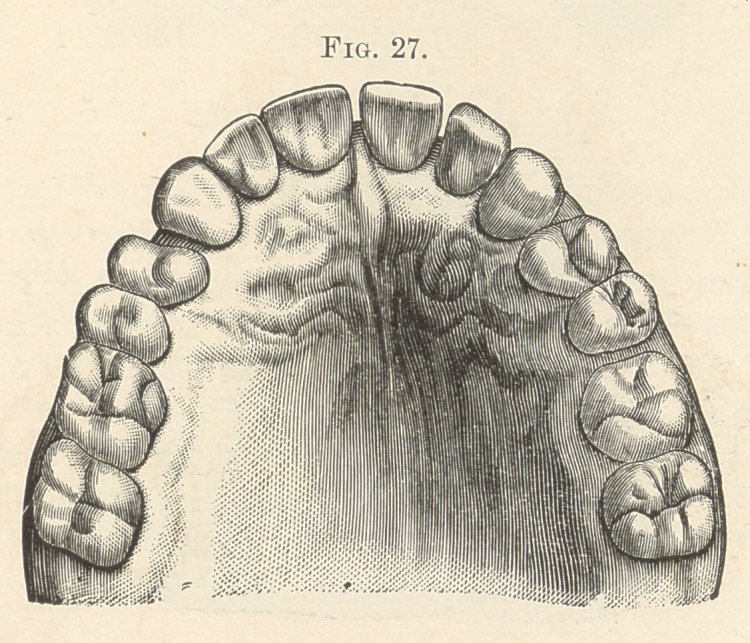


**Fig. 28. f18:**
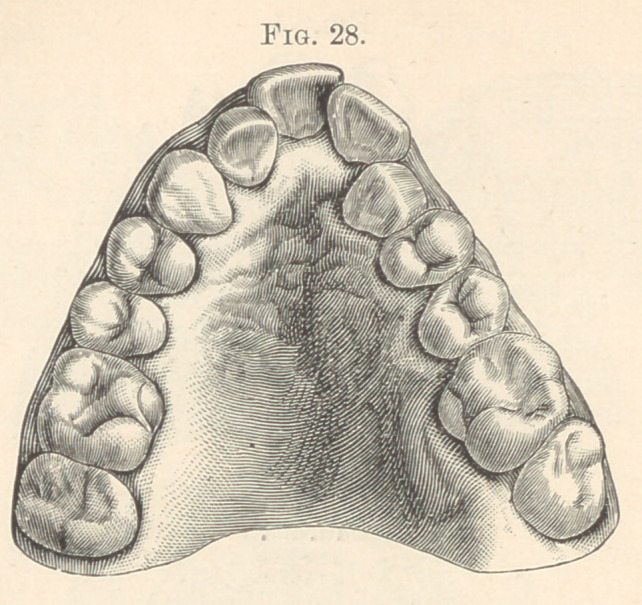


**Fig. 29. f19:**
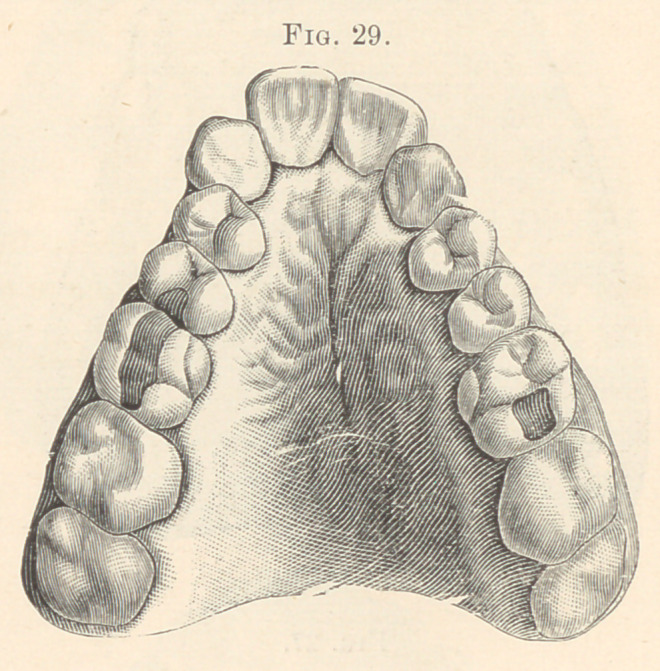


**Fig. 30. f20:**